# Pneumocephalus as a Rare Presentation of Acute Sinusitis with Intracranial Extension – Two Cases and a Review of Literature

**DOI:** 10.25259/JCIS-26-2019

**Published:** 2019-06-14

**Authors:** Xingwen Sun, Gloria C. Chiang

**Affiliations:** Department of Radiology, Weill Cornell Medical College, New York-Presbyterian Hospital, New York, United States.

**Keywords:** Sinusitis, Meningitis, Pneumocephalus, Imaging

## Abstract

Pneumocephalus is a rare presentation of sinusitis. The purpose of this case series and literature review is to highlight the critical importance of recognizing pneumocephalus on initial brain imaging, since it may serve as the first sign of acute sinusitis with intracranial complication. Although sinusitis is a treatable condition with appropriate and timely antibiotic therapy, failure to recognize intracranial extension and delays to prompt surgical drainage could lead to a dire clinical course.

## INTRODUCTION

Pneumocephalus refers to the presence of air within the intracranial compartment, which occurs typically from trauma or surgery. Pneumocephalus is rare in the setting of infection without a breach in the calvarium, accounting for only 1% of cases in a historic case series,^[1]^ with the majority attributable to otomastoiditis. The purpose of this paper is to review the current imaging literature to characterize the clinical symptoms, neuroanatomical considerations, inciting pathogens, and clinical course of patients presenting with pneumocephalus in the setting of sinusitis. We reviewed the literature by searching MEDLINE through the United States National Library of Medicine’s PubMed online database, using the keywords: Pneumocephalus, sinusitis, meningitis, and infection. Relevant case reports, reviews, and their references were also examined for further sources. For each, we documented the clinical presentation, location of the sinusitis and pneumocephalus, whether imaging identified an osseous defect, and the subsequent clinical course. We further present in detail two additional cases of patients with bacterial sinusitis presenting with pneumocephalus. These cases and the accompanying review aim to highlight the critical importance of recognizing pneumocephalus as a rare presentation of sinusitis, since it may serve as the first sign of an intracranial complication. Although bacterial sinusitis is a treatable condition with appropriate and timely antibiotic therapy, failure to recognize intracranial extension and delays to prompt surgical drainage could lead to a dire clinical course.

## CASE REPORTS

### Case 1

A 62-year-old woman presented with headache, nasal congestion, and vertigo. She presented to her primary care physician and was treated with antibiotics for presumed sinusitis. After days of having no improvement in her condition, she presented to the emergency department, where she was found to be mildly confused and agitated, not answering questions appropriately.

**Figure 1:**
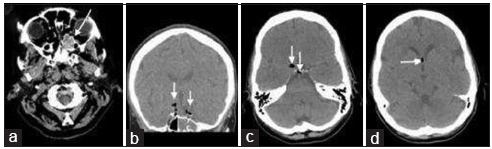
A 62-year-old woman presenting with headache and vertigo found to have a mucopyocele and pneumocephalus. (a) Non-contrast-enhanced axial CT image of the head demonstrates hyperattenuation within an expanded left posterior ethmoid air cell (arrow), initially considered a chronic mucocele with inspissated secretions, but later found to be a mucopyocele intraoperatively. (b) Coronal CT reformation and (c,d) axial non-contrast CT images of the head demonstrate foci of pneumocephalus (arrows) in the parasellar region, suprasellar cistern, and cavum septum pellucidum, suspicious for an intracranial extension.

A non-contrast computerized tomography (CT) scan of the head [Figure 1] showed complete opacification and expansion of a left posterior ethmoid air cell with hyperattenuating material reported to be a mucocele with chronic inspissated secretions. No paranasal sinus air-fluid levels were identified, and thin-slice reformations with a bone algorithm demonstrated no osseous defect or erosion. However, foci of pneumocephalus were seen in the suprasellar cistern, parasellar region, and cavum septum pellucidum, raising the possibility of a suspicious intracranial process.

**Figure 2:**
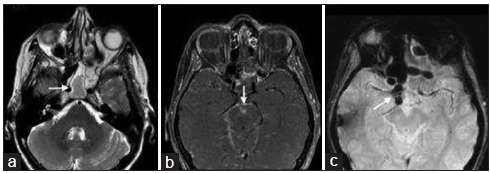
A 62-year-old woman presenting with headache and vertigo found to have meningitis, secondary to the intracranial extension from sinusitis. (a) T2-weighted axial MRI image through the brain demonstrates T2 hyperintensity within the left sphenoid and ethmoidal air cells (arrow). (b) Contrast-enhanced axial MRI image of the brain demonstrates leptomeningeal enhancement (arrow) in the interpeduncular fossa and along the cerebral peduncles, consistent with meningitis. (c) Susceptibility-weighted MRI image demonstrates round foci of susceptibility hypointensity in the parasellar region with blooming artifact (arrow), consistent with pneumocephalus.

A magnetic resonance imaging (MRI) scan of the brain was then performed [Figure 2] which demonstrated basilar meningitis, with leptomeningeal enhancement in the interpeduncular fossa, as well as along the ventral midbrain, pons, and medulla. Lumbar puncture revealed cloudy cerebrospinal fluid (CSF) with an elevated white blood cell count (262/μl, normal 0–5/μl) with 75% neutrophils, consistent with bacterial meningitis. Blood cultures were positive for hemophilus influenzae.

Urgent surgical debridement was performed, with drainage of purulent material from the left sphenoid sinus. The previously reported mucocele in the left posterior ethmoid sinus was found to be a mucopyocele intraoperatively. The patient recovered well after a subsequent course of intravenous antibiotics.

### Case 2

A 9-year-old boy presented with a 1-week history of frontal headaches, with intermittent cough and rhinorrhea. Medical history was unremarkable, including a normal birth history and developmental milestones. On the day of admission, he was found to have a fever of 104°F (40°C), and the family reported a possible seizure.

**Figure 3:**
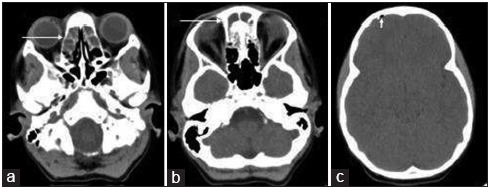
A 9-year-old boy presenting with headache and vertigo found to have frontal sinusitis and pneumocephalus. (a-c) Axial non-contrast CT images of the head demonstrate fluid attenuation within the bilateral ethmoid sinuses, frontal sinuses, and a small right frontal focus of pneumocephalus (arrows).

Non-contrast CT of the head demonstrated opacification of the ethmoid and frontal sinuses. The remainder of the head CT was reported to be normal. The patient demonstrated no clinical improvement and was transferred to our institution for further management. Neuroradiological consultation identified a small focus of pneumocephalus in the right frontal extra-axial space, which was initially overlooked [Figure 3]. Thin-slice reformations through the sinuses failed to reveal an osseous defect or erosion.

**Figure 4:**
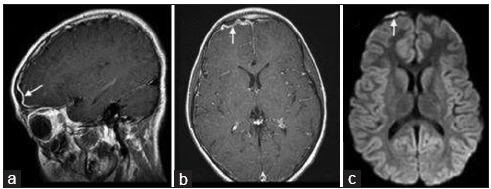
A 9-year-old boy with frontal sinusitis and pneumocephalus found to have an epidural abscess. (a) Sagittal and (b) axial gadolinium-enhanced T1-weighted MR images of the brain demonstrate a contrast-enhancing fluid collection (arrow) overlying the right frontal lobe. (c) The diffusion-weighted image demonstrates restricted diffusion, compatible with an epidural abscess (arrow).

MRI of the brain was then performed and demonstrated a right frontal epidural fluid collection, most compatible with an epidural abscess [Figure 4]. Leptomeningeal enhancement underlying the abscess in the right frontal lobe was compatible with meningitis.

Lumbar punctures revealed CSF with slightly elevated white blood cell count (24/μl and 19/μl, normal 0–5/μl) with neutrophilic predominance (91%) indicating bacterial meningitis. The patient underwent emergent functional endoscopic sinus surgery and supraorbital craniotomy for epidural abscess drainage and debridement. The culture of the abscess grew coagulase-negative *Staphylococcus aureus* and *Escherichia coli*. The patient was discharged home with intravenous antibiotics.

## DISCUSSION

Pneumocephalus as a presenting symptom of sinusitis is rare. In a historical review of 295 cases of pneumocephalus, before the widespread use of cross-sectional imaging, trauma was reported as the most common cause, accounting for 75% of cases.^[1]^ On the other hand, infection is a rare cause of pneumocephalus and is typically otogenic when it occurs.^[1]^

Although sinusitis is often considered a common and benign entity due to the widespread and early use of antibiotics, there is still a reported 4% incidence of intracranial complication.^[2]^ While uncomplicated sinusitis may be symptomatically managed or treated by oral antibiotics, surgical debridement must be initiated promptly when there is an intracranial extension.^[2]^ Missing subtle early signs of the intracranial extension, as in Case 2, could delay proper management and severe neurological damage may occur.

In our review of the literature [Table 1], we found a total of 8 cases of pneumocephalus in the setting of sinusitis.^[3-10]^ Two cases were reported before the era of antibiotics and imaging.^[3,4]^ In both cases, chronic CSF rhinorrhea suggested significant osseous erosion by the time of presentation, and both patients died from their intracranial complications. Of the more recent 6 cases, the only two patients who died had fungal sinusitis as the inciting process.^[5,6]^ Half of the cases reported a visible osseous defect on CT imaging.^[6-8]^ However, as seen with our 2 cases, neither osseous erosion nor fungal infection is necessary for the development of pneumocephalus and intracranial extension. Several patients previously reported also had significant neurological deficits or meningeal signs that raised the clinical suspicion for an intracranial extension,^[5,8,9]^ whereas our two patients reported only mild symptoms of headache and fever and had no concerning clinical signs on initial examination. As a result, the pneumocephalus on non-contrast CT of the head was the only indication of a potentially worrisome clinical course.

**Table 1:** Summary of 8 cases of pneumocephalus in sinusitis from literature review.

Author Year	Age Sex	Presenting symptoms	Origin of infection	Location of pneumocephalus	Presence of bony defect	Pathogen	Clinical course
Louis 1927^[4]^	11 Male	Spontaneous CSF rhinorrhea	Frontal	Frontal epidural and intraventricular	No, the only radiograph performed		Sinusotomy Brain abscess, Death
Plunkett 1935^[3]^	54 Male	Paresis, CSF rhinorrhea	Ethmoid	Intraventricular	No, the only radiograph performed		Died, Brain abscess at autopsy
Naganuma 1986^[7]^	18 Male	Headache Fever	Sphenoid	Basilar cisterns	Yes, roof of the sphenoid	Klebsiella	None
Winton 1987^[9]^	57 Female	Headache Fever Altered mental status	Sphenoid	Not reported	No	Strep pneumo	Meningitis, IV abx discharged
Engel 2000^[5]^	77 Male	Altered mental status	Frontal	Frontal epidural	No	Aspergillus	Immunosuppressed, death
Apostolakos 2007^[10]^	61 Male	Headache Fever	Sphenoid	Basilar cisterns	Unknown	Strep pneumo	Meningitis, no follow-up
Lin 2009^[6]^	14 Male	Headache Fever	Sphenoid	Basilar cisterns	Yes, clivus	Candida	Meningitis, cavernous thrombosis, death
Ohe 2012^[8]^	60 Male	Altered mental status	Sphenoid	Basilar cisterns	Yes, clivus	Strep pneumo	Endonasal drainage, IV abx, bedridden from meningitis

CSF: Cerebrospinal fluid

In most cases, the location of the pneumocephalus corresponded to the diseased paranasal sinus in closest proximity. Our first patient was found to have pneumocephalus in the basilar cisterns, in close proximity to the patient’s sphenoid and posterior ethmoid sinusitis. The location of the pneumocephalus corresponded to purulent material associated with meningitis on subsequent MRI. Many have reported osseous defects in the skull base, which could precipitate the intracranial extension of a sinus infection. Although our patient did not have a detectable osseous defect on CT or during surgery, the lateral sphenoid region may harbor congenital fistulae that would predispose to an intracranial extension.^[11]^ Coughing and sneezing could result in enough pressure to cause a “ball-valve” effect through small fistulae,^[12]^ introducing air, and pathogens into the intracranial compartment.

Small air collections can be difficult to identify. In our second case, the single focus of pneumocephalus in the right frontal epidural space was initially overlooked. On further review, the possibility of intracranial extension and epidural abscess formation from the frontal sinuses was considered. Besides well-established antibiotic treatment regimens, epidural abscess associated with frontal sinusitis is increasingly reported.^[13]^ Patients with frontal sinusitis may have subclinical presentations, which often lead to a delayed diagnosis. Complications such as subperiosteal abscess and osteomyelitis of the frontal calvarium are frequently identified weeks after the onset, when an abscess becomes more visible on CT or the patient reports severe clinical symptoms.^[14]^ In our case, the subtle focus of air on initial CT was the key to timely management.

## CONCLUSION

We present these two cases and a literature review to highlight the importance of identifying pneumocephalus as the first sign of intracranial complication in the setting of bacterial sinusitis. Neither osseous erosion nor fungal infection is necessary to produce pneumocephalus, and, although rare, pneumocephalus may be the key to timely diagnosis and management of these patients.

## References

[1] Markham JW (1967). The clinical features of pneumocephalus based upon a survey of 284 cases with report of 11 additional cases. Acta Neurochir (Wien).

[2] Clayman GL, Adams GL, Paugh DR, Koopmann CF (1991). Intracranial complications of paranasal sinusitis: A combined institutional review. Laryngoscope.

[3] Plunkett JE, Lendrum FC (1935). A case of spontaneous pneumocephalus and cerebrospinal rhinorrhoea. Can Med Assoc J.

[4] Louis A, Levison MD (1927). Spontaneous ventriculography from ruptured brain abscess. JAMA.

[5] Engel G, Fearon WF, Kosek JC, Loutit JS (2000). Pneumocephalus due to invasive fungal sinusitis. Clin Infect Dis.

[6] Lin JJ, Wu CT, Hsia SH, Wang HS, Lin KL (2009). Pneumocephalus: A rare presentation of candida sphenoid sinusitis. Pediatr Neurol.

[7] Naganuma H, Imai S, Wakao T, Koizumi H, Koizumi J, Minowa A (1986). Pneumocephalus secondary to acute sinusitis case report. Neurol Med Chir (Tokyo).

[8] Ohe Y, Maruyama H, Deguchi I, Fukuoka T, Kato Y, Nagoya H (2012). An adult case of pneumocephalus and pneumococcal meningitis associated with the sphenoid sinusitis. Intern Med.

[9] Winton MD, Everett ED, Watts CH (1987). Pneumococcal infection and gurgling in the head. Ann Intern Med.

[10] Apostolakos D, Roistacher K (2007). Pneumocephalus. Mayo Clin Proc.

[11] Lefranc M, Peltier J, Demuynkc F, Bugnicourt JM, Desenclos C, Fichten A (2009). Tension pneumocephalus and rhinorrhea revealing spontaneous cerebrospinal fluid fistula of the anterior cranial base. Neurochirurgie.

[12] Rabello FA, Massuda ET, Oliveira JA, Hyppolito MA (2013). Otogenic spontaneous pneumocephalus: Case report. Braz J Otorhinolaryngol.

[13] Nicoli TK, Mäkitie A (2014). Images in clinical medicine. Frontal sinusitis causing epidural abscess and puffy tumor. N Engl J Med.

[14] Palabiyik FB, Yazici Z, Cetin B, Celebi S, Hacimustafaoglu M (2016). Pott puffy tumor in children: A Rare emergency clinical entity. J Craniofac Surg.

